# miR-221/222 Profiles in Small Extracellular Vesicles Derived from Gemcitabine-Sensitive and -Resistant A549 Cancer Stem-like Cell Spheroids: An In Vitro Study

**DOI:** 10.3390/biomedicines14102232

**Published:** 2026-10-02

**Authors:** Aynaz Ghorbani, Onur Uysal, Dilay Karacaoğlu

**Affiliations:** 1 Department of Stem Cell, Institute of Health Sciences, Eskisehir Osmangazi University, 26040 Eskisehir, Türkiye; 2 Cellular Therapy and Stem Cell Production Application and Research Centre, ESTEM, Eskisehir Osmangazi University, 26040 Eskisehir, Türkiye

**Keywords:** non-small cell lung cancer, A549, gemcitabine resistance, cancer stem-like cells, spheroid culture, small extracellular vesicles, miR-221/222, MDR1

## Abstract

**Background:** Gemcitabine is used in the treatment of human non-small cell lung cancer (hNSCLC), yet acquired resistance can limit therapeutic responses. Small extracellular vesicles (sEVs) and microRNAs (miRNAs) have been implicated in chemoresistance-associated intercellular signaling. We profiled the miR-221/222 cluster and resistance-associated genes in cancer stem-like cells (CSLCs) and CSLC-derived sEV preparations from gemcitabine-sensitive and -resistant A549 models. **Methods:** Gemcitabine-resistant A549 cells and matched cancer stem-like cell (CSLC) and gemcitabine-resistant cancer stem-like cell (Gr-CSLC) populations were generated and characterized by flow cytometry, qRT-PCR, and immunofluorescence. sEV preparations derived from CSLC and Gr-CSLC spheroids were characterized for total protein content, EV-associated tetraspanins, and vesicular morphology. PKH26-associated fluorescence following exposure of A549 cells to labeled sEV preparations was also evaluated. The relative levels of sEV-associated miR-221/222 family members and the resistance-associated genes MDR1, ABCG2, and BCL2 were assessed by qRT-PCR. **Results:** Gr-CSLCs exhibited enhanced spheroid formation and increased MDR1 expression. Gr-CSLC-derived sEV preparations showed higher total protein content than CSLC-derived preparations. Bead-based flow cytometry demonstrated EV-associated CD9 and CD81 expression, while cryo-TEM revealed small, membrane-bound vesicular structures. PKH26-associated intracellular fluorescence was observed in A549 cells following exposure to labeled sEV preparations. Gr-CSLC-derived sEVs showed higher relative levels of miR-221-3p, miR-222-3p, and miR-222-5p than CSLC-derived sEVs, whereas miR-221-5p showed no significant difference. MDR1 and BCL2 also showed higher relative expression in Gr-CSLC-derived sEVs, whereas ABCG2 showed no significant difference. **Conclusions:** sEV preparations derived from gemcitabine-resistant Gr-CSLCs exhibited distinct miR-221/222 and resistance-associated RNA profiles compared with those derived from gemcitabine-sensitive CSLCs in this in vitro A549 NSCLC model. These molecular differences should be interpreted as associations with the resistant phenotype rather than evidence of functional transfer of chemoresistance. Further functional studies and validation in additional NSCLC and patient-derived models are required to establish their biological, therapeutic, or biomarker relevance.

## 1. Introduction

Lung cancer remains one of the most commonly diagnosed malignancies and a leading cause of cancer-related mortality worldwide [1]. Human non-small cell lung cancer (hNSCLC) accounts for approximately 85% of lung cancer cases and continues to pose major therapeutic challenges despite advances in early detection and multimodal treatment [2]. Gemcitabine (2′,2′-difluorodeoxycytidine, dFdC), a nucleoside analog that is incorporated into replicating DNA, is widely used in hNSCLC chemotherapy regimens [3]. However, the clinical benefit of gemcitabine is frequently limited by the emergence of acquired chemoresistance, particularly following relapse and metastatic progression [4].

The tumor microenvironment comprises stromal and immune cells together with a minor population of cancer stem cells (CSCs). CSCs display self-renewal capacity, multilineage differentiation potential, and enhanced survival, and they are increasingly recognized as key drivers of tumor initiation, therapeutic resistance, recurrence, and metastasis [5,6]. Although CSC–microenvironment crosstalk is known to contribute to treatment failure, the molecular mediators that enable resistance to persist and disseminate across heterogeneous tumor cell populations remain only partially elucidated [7]. Three-dimensional spheroid culture provides a useful in vitro approach for enriching cancer stem-like (CSL) populations and evaluating CSC-associated characteristics under conditions that differ from conventional two-dimensional monolayer culture [8]. Spheroid-based models are also increasingly used in extracellular vesicle research because three-dimensional growth conditions can influence tumor-cell interactions and extracellular vesicle-associated biological features [9].

Exosomes have emerged as important mediators of intercellular communication in cancer [10]. These small extracellular vesicles (sEVs) (typically 70–140 nm) are released by many cell types and can be internalized by neighboring or distant cells, thereby transferring proteins, DNA, mRNAs, miRNAs, and long non-coding RNAs [11,12]. Through their cargo, exosomes influence angiogenesis, immune evasion, invasion, metastasis, and treatment response [13]. Among exosomal cargos, miRNAs exert potent post-transcriptional regulatory functions. Oncogenic miRNAs (oncomiRs) can repress tumor suppressor pathways and promote malignant phenotypes, whereas tumor-suppressive miRNAs can restrain tumor progression [14,15,16]. Consequently, exosomal miRNA profiles are being intensively explored as candidate biomarkers and mechanistic readouts of tumor behavior and therapy response, particularly in controlled in vitro systems [17,18].

The miR-221/222 cluster is among the most extensively studied oncomiR families and is overexpressed in several malignancies, including lung cancer [19]. These miRNAs have been implicated in tumor-promoting processes through the regulation of multiple targets, including PTEN and ERα, thereby influencing cell proliferation, migration, invasion, and apoptosis [20,21]. Context-dependent tumor-suppressive functions have also been reported, such as the inhibitory effect of miR-222 on SIRT1-mediated signaling in prostate cancer [22]. Despite this growing body of evidence, the involvement of the miR-221/222 cluster in gemcitabine resistance in A549-derived models—particularly within CSC populations and their spheroid-derived small extracellular vesicles (sEVs)—remains incompletely characterized.

Accumulating data indicate that tumor-derived exosomes can propagate chemoresistance traits horizontally among heterogeneous tumor cell populations. Because resistance may arise within a CSC subpopulation and subsequently influence bulk tumor cells, exosomal miRNAs represent plausible mediators of this process. Building on our previously established and phenotypically validated gemcitabine-resistant cancer stem-like cell (CSLC) model [23], we hypothesized that miR-221/222 profiles in sEVs released from gemcitabine-resistant CSLCs reflect the resistant phenotype of their parental cells and may be associated with resistance-related molecular features. Therefore, this study aimed to examine the expression of miR-221-3p, miR-221-5p, miR-222-3p, and miR-222-5p in CSLCs isolated from gemcitabine-sensitive and -resistant A549 cell lines and in their secreted sEVs, together with resistance-associated genes (MDR1, ABCG2, BCL2) and pluripotency-related markers (SOX2, OCT4, NANOG). In addition, we emphasize a spheroid-based, cell culture–compatible workflow for generating matched CSLC/Gr-CSLC populations and profiling their sEV-associated RNA cargo under defined in vitro conditions.

## 2. Materials and Methods

### 2.1. Cell Models

In our previous work [23], a gemcitabine-hydrochloride–resistant A549 (Gr-A549) hNSCLC model was generated from parental A549 cells (ATCC® CCL-185™, Manassas, VA, USA) by stepwise exposure to increasing gemcitabine concentrations over 15 months. Because the present study focuses on the post-thaw characterization of CSLC/Gr-CSLC populations and their derived small extracellular vesicles (sEVs), previously reported quantitative dose–response estimates, including exact IC_50_ values and resistance-fold calculations, were not used as outcome measures in the current analysis. Subsequently, CSLCs and Gr-CSLCs were isolated through sequential CD326+, CD133+, and CD44+ immunoselection using the S-pluriBead® Maxi Reagent Kit (Cat. No. 70-50010-12; pluriSelect Life Science, Leipzig, Germany). The isolated CSLCs were comprehensively characterized by flow cytometry and immunofluorescence for CD326, CD133, CD44, CD90, ABCG2 and MDR1, and evaluated for tumorsphere formation in 3D culture (agarose, PDMS, PolyHEMA), and gemcitabine sensitivity via MTT assay. All characterized CSLC and Gr-CSLC spheroids were cryopreserved under validated conditions. In the current study, previously characterized CSLC and Gr-CSLC lines were thawed and cultured under serum-free spheroid conditions to enable isolation and molecular characterization of their secreted sEVs.

### 2.2. Thawing and Culture of CSLC and Gr-CSLC Spheroids

Cryopreserved CSLC and Gr-CSLC spheroids were thawed rapidly in a 37 °C water bath for ~2 min until a small ice pellet remained. The contents of each cryovial were transferred aseptically into 15 mL conical tubes containing 10 mL of pre-warmed RPMI 1640 medium (Capricorn Scientific GmbH, Ebsdorfergrund, Germany) and centrifuged at 300× *g* for 5 min to remove DMSO. The cell pellets were gently resuspended in serum-free CSLC culture medium, composed of RPMI 1640 supplemented with 1% MEM Nonessential Amino Acids (NEAA-B; Capricorn Scientific GmbH, Ebsdorfergrund, Germany), 20 ng/mL recombinant human EGF (Cat. No. 585506; BioLegend, San Diego, CA, USA), 20 ng/mL recombinant human FGF (Cat. No. 571506; BioLegend, San Diego, CA, USA), and penicillin–streptomycin (Capricorn Scientific GmbH, Ebsdorfergrund, Germany). Equal numbers of Gr-CSLCs and CSLCs (2 × 10^4^ cells/well) were seeded into 6-well ultra-low attachment plates (Corning Inc., Corning, NY, USA) in a total volume of 2 mL per well. Cultures were maintained at 37 °C, 5% CO_2_, 5% O_2_, and medium was refreshed every 48 h. Spheroid formation was monitored daily under a phase-contrast inverted microscope (Axio Observer D1, Carl Zeiss Microscopy GmbH, Oberkochen, Germany). Spheroids were considered mature and morphologically viable when each spheroid consisted of at least 50–60 cells and displayed no central dark zones or necrotic cores. At this stage, approximately 15–20 uniform spheroids per well were observed. When total viable cell count reached approximately 5 × 10^5^ cells per well, sEV isolation procedures were initiated under equal culture conditions.

### 2.3. Viability and Immunophenotypic Analyses of Thawed CSLC and Gr-CSLC Lines

To verify that the freeze–thaw process did not compromise viability or stemness, cryopreserved CSLC and Gr-CSLC spheroids were re-evaluated. Cell viability and total cell numbers were determined using the Trypan Blue Exclusion Assay (Gibco™, Thermo Fisher Scientific, Waltham, MA, USA) and counted in a Neubauer hemocytometer under light microscopy (Zeiss Axio Observer D1, Carl Zeiss Microscopy GmbH, Oberkochen, Germany).

Immunophenotypic profiling by flow cytometry was performed to confirm retention of CSLC-specific surface markers after thawing. Cells were stained with monoclonal antibodies against CD326 (EpCAM), CD133, CD44, CD90, ABCG2 (CD338), ALDH1, and MDR1 (ABC transporter) to assess stemness and differential drug resistance between CSLC and Gr-CSLC populations. All antibodies (BioLegend, San Diego, CA, USA) were used in the following conjugated forms: CD133-PE/Cyanine7, CD44-FITC, CD90-FITC, ABCG2-FITC, MDR1-Alexa Fluor^®^ 488, and purified ALDH1. Analyses were performed on a Novocyte D3005 flow cytometer (Agilent Technologies, Santa Clara, CA, USA), and data were processed using NovoExpress v1.5.0 software (Agilent), with events gated on forward and side scatter parameters and corresponding isotype controls. To assess stemness-associated transcription factors, SOX2, OCT4 and NANOG expression was quantified by qRT-PCR in CSLC and Gr-CSLC spheroids.

### 2.4. Isolation and Characterization of CSLC- and Gr-CSLC-Derived Small Extracellular Vesicles

#### 2.4.1. Small Extracellular Vesicle (sEV) Isolation

After 72 h of serum-free culture, conditioned media from CSLC and Gr-CSLC spheroids were collected, and sEVs were isolated by sequential differential centrifugation and ultracentrifugation. Supernatants were centrifuged at 300× *g* for 10 min to remove floating cells and at 3000× *g* for 30 min to eliminate cellular debris, then filtered through a 0.22 µm syringe filter to exclude larger vesicles and apoptotic bodies. The filtrate was ultracentrifuged at 100,000× *g* for 120 min at 4 °C using an Optima XPN-100 ultracentrifuge (Beckman Coulter Life Sciences, Indianapolis, IN, USA), and the resulting sEV-enriched pellet was centrifuged again at 100,000× *g* for 70 min. Final sEV-enriched pellets were resuspended in 100 µL Dulbecco’s phosphate-buffered saline (DPBS; Capricorn Scientific GmbH, Ebsdorfergrund, Germany) and stored at −80 °C until use.

#### 2.4.2. Characterization of CSLC- and Gr-CSLC-Derived sEVs

Protein Quantification: Total protein content of sEV preparations from CSLC and Gr-CSLC cultures was quantified using the Qubit™ Protein Assay Kit and Qubit™ 4 Fluorometer (Invitrogen™, Thermo Fisher Scientific, Waltham, MA, USA) according to the manufacturer’s instructions. sEV suspensions resuspended in 100 µL sterile DPBS were lysed in Pierce™ RIPA lysis buffer (Thermo Fisher Scientific, Waltham, MA, USA). A standard curve was generated using bovine serum albumin (BSA) standards supplied with the kit. Ten microliters of each sEV lysate were mixed with 190 µL working solution in Qubit assay tubes, incubated for 15 min at room temperature in the dark, and measured on the fluorometer. Each sample was analyzed in triplicate; protein concentrations (µg/mL) were normalized across groups for downstream applications, and sEV samples were aliquoted and stored at −80 °C until further use.

sEV Surface Marker Analysis by Flow Cytometry: Expression of the EV-associated tetraspanins CD9, and CD81 were assessed in sEV preparations derived from CSLC and Gr-CSLC cultures by bead-based flow cytometry. sEV preparations (10 µg total protein) were captured with CD63-coated magnetic beads (Miltenyi Biotec, Bergisch Gladbach, Germany) in PBS containing 0.1% BSA (Capricorn Scientific, Ebsdorfergrund, Germany) for 2 h at room temperature under gentle rotation (15 rpm), then washed twice with PBS. Bead-bound sEVs were stained for 30 min at 4 °C in the dark with CD9-PE (phycoerythrin), CD81-FITC (fluorescein isothiocyanate), and CD63-APC (allophycocyanin) monoclonal antibodies (BioLegend, San Diego, CA, USA) using the manufacturer’s recommended dilutions; matched fluorochrome-conjugated isotype controls were included to assess nonspecific binding. After staining and washing, samples were resuspended in 500 µL PBS and analyzed on a Novocyte D3005 flow cytometer (Agilent Technologies, Santa Clara, CA, USA). Detector voltages were set to 497 V for FITC and 565 V for PE and APC, and spectral compensation was applied automatically using NovoExpress v1.5.0 software (Agilent). Events were gated on forward and side scatter properties characteristic of bead–sEV complexes, with a minimum of 10,000 gated events per sample. All analyses were performed in biological triplicates (*n* = 3), and the percentage of positive events was determined for each marker.

Cellular Uptake of CSLC- and Gr-CSLC-Derived sEVs by A549 Cells: To evaluate the internalization of CSLC- and Gr-CSLC-derived sEV preparations, A549 human non-small cell lung carcinoma (hNSCLC) cells were used as recipient cells. A fluorescence-based tracking assay was performed using the PKH26 Red Fluorescent Cell Linker Kit (Sigma-Aldrich, Darmstadt, Germany), a lipophilic fluorescent dye used to label lipid-containing vesicular preparations for fluorescence-based tracking. 

Isolated sEV preparations (20 µg total protein) were diluted in 80 µL Diluent C, incubated with 1 µL PKH26 for 5 min at room temperature with gentle mixing, quenched with 2 mL 0.5% bovine serum albumin (BSA; Sigma-Aldrich, Darmstadt, Germany) in PBS, and ultracentrifuged at 100,000× *g* for 70 min at 4 °C to recover the labeled sEV preparation. Pellets were resuspended in 200 µL sterile PBS. For uptake assays, 1 × 10^5^ A549 cells (ATCC® CCL-185™, Manassas, VA, USA) were seeded in 6-well plates and cultured in RPMI 1640 (Gibco™, Thermo Fisher Scientific, Waltham, MA, USA) supplemented with 10% FBS and 1% penicillin–streptomycin. After overnight adherence, the medium was replaced with serum-free RPMI, and PKH26-labeled sEV preparations were added (final concentration equivalent to 10 µg total sEV-associated protein/mL). Following overnight incubation at 37 °C, 5% CO_2_, cells were washed three times with PBS, fixed with 4% paraformaldehyde for 15 min, and nuclei were counterstained with Hoechst 33342 (Thermo Fisher Scientific, Waltham, MA, USA) for 10 min. Uptake was visualized using a Zeiss Axio Observer D1 fluorescence microscope (Carl Zeiss Microscopy GmbH, Oberkochen, Germany) equipped with appropriate filter sets. Images were acquired with a Zeiss AxioCam MRm camera and processed using ZEN Imaging Software v3.12 (Carl Zeiss Microscopy GmbH, Oberkochen, Germany). Uptake-associated fluorescence was defined as intracellular red PKH26-associated signals localized predominantly in the perinuclear cytoplasm of Hoechst-stained A549 cells. All experiments were performed in triplicate.

Morphological Analysis of sEVs by Cryo-TEM: Ultrastructural morphology and diameter of sEVs isolated from CSLC and Gr-CSLC cultures were evaluated by cryogenic transmission electron microscopy (cryo-TEM) to preserve native architecture and avoid dehydration- or staining-related artifacts. sEV suspensions were diluted in sterile PBS to ~100 µg/mL, and 3–4 µL of each sample was applied onto carbon-coated holey copper grids (Quantifoil^®^ R2/2, 300 mesh; Quantifoil Micro Tools GmbH, Großlöbichau, Germany) previously glow-discharged with a PELCO easiGlow™ Glow Discharge Cleaning System (Ted Pella, Inc., Redding, CA, USA) to enhance hydrophilicity. Grids were vitrified by rapid immersion in liquid ethane using a Vitrobot Mark IV (Thermo Fisher Scientific, Waltham, MA, USA) at 4 °C and 100% humidity, then imaged at −178 °C on a Hitachi HT7800 transmission electron microscope (Hitachi High-Tech Corporation, Tokyo, Japan) operated at 100 kV. Images were captured with a CCD camera (Hitachi High-Tech Corporation, Tokyo, Japan) at 20,000–80,000× magnification without negative staining, and quantitative diameter measurements were obtained for at least 10 individual vesicular structures per group using the integrated imaging software.

### 2.5. qRT-PCR Analysis

Total RNA was isolated from A549-derived cancer stem like cell (CSLC) and gemcitabine-resistant CSLC (Gr-CSLC) spheroids and their corresponding small extracellular vesicles (sEVs) to evaluate the expression of drug-resistance-related genes. RNA from spheroids and sEV preparations was extracted using the miRNeasy Micro Kit and exoRNeasy Serum/Plasma Kit, respectively (Qiagen, Hilden, Germany), according to the manufacturers’ instructions. RNA purity and concentration were assessed using a NanoDrop™ One spectrophotometer (Thermo Fisher Scientific, Waltham, MA, USA). The total RNA input was standardized to 100 ng for each sample prior to reverse transcription. cDNA for mRNAs was synthesized using the QuantiTect Reverse Transcription Kit, whereas cDNA for miRNAs was synthesized using the miRCURY LNA RT Kit (Qiagen, Hilden, Germany). Expression of miR-221-3p, miR-221-5p, miR-222-3p, and miR-222-5p, as well as the mRNA targets BCL2, MDR1 (ABCB1), and ABCG2, was analyzed in CSLCs, Gr-CSLCs, and their corresponding sEV preparations. To confirm stemness characteristics, SOX2, OCT4, and NANOG were quantified only in CSLC and Gr-CSLC spheroids and were not analyzed in sEV samples. qRT-PCR was performed using the QuantiTect SYBR Green PCR Kit on a Rotor-Gene Q system (Qiagen, Hilden, Germany). Reactions were performed in biological triplicates, with an initial denaturation at 95 °C for 10 min, followed by 40 cycles of 95 °C for 20 s, 60 °C for 30 s, and 72 °C for 30 s. U6 snRNA and GAPDH served as internal reference genes for miRNAs and mRNAs, respectively. To monitor potential contamination of the qRT-PCR reagents, at least one no-template control, in which cDNA template was omitted, was included for each gene and sample set. Relative gene expression was calculated using the 2^−^ΔΔCt method. The stability of U6 and GAPDH across cellular and sEV samples was not independently validated in the present study; therefore, comparisons of relative RNA abundance between cellular and sEV compartments should be interpreted with caution. For the miRNA assays, the target-specific sequences listed in Table 1 were used together with the common reverse-primer sequence (5′-CGAGGAAGAAGACGGAAGAAT-3′) according to the original experimental protocol. Independent primer-efficiency validation data were not available for retrospective analysis. Primer sequences are provided in Table 1.

### 2.6. Statistical Analysis

All experiments and reactions were performed in biological triplicates (*n* = 3), representing three independent cell culture experiments. For sEV analyses, sEVs were isolated separately from each independent culture, resulting in three independent sEV isolations. Technical replicates, where applicable, were used only for assay-level measurements and were not considered independent biological replicates for statistical analysis. Data were analyzed by one-way analysis of variance (ANOVA) followed by Tukey’s honestly significant difference (HSD) post hoc test. Results are expressed as mean ± standard error of the mean (SEM), and *p* < 0.05 was considered statistically significant. Statistical analyses were performed using IBM SPSS Statistics v13.0 (IBM Corp., Armonk, NY, USA).

## 3. Results

**Figure 1:**
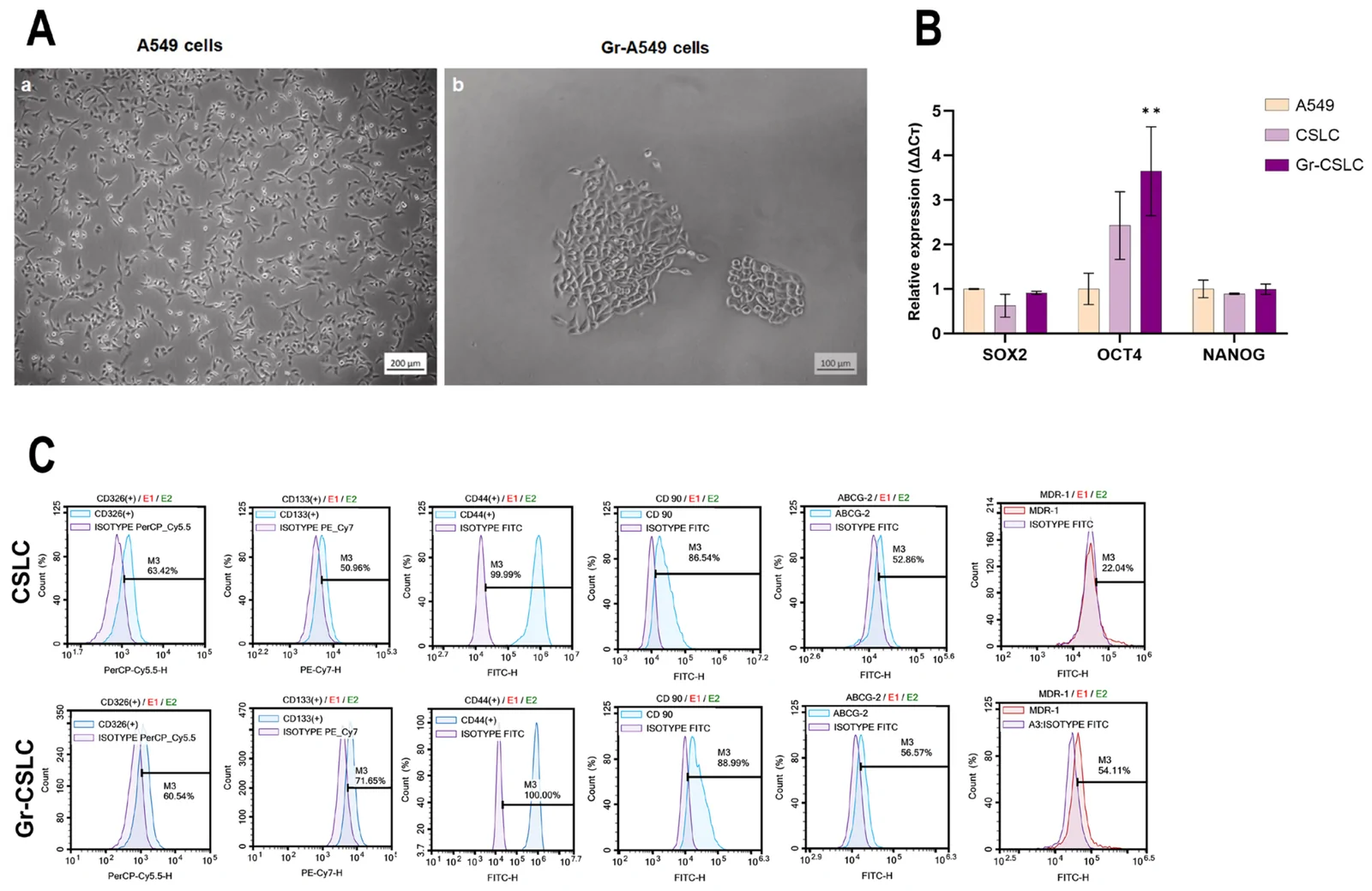
In vitro characterization of CSLCs and Gr-CSLCs. (**A**): Morphology of (**a**) A549 and (**b**) Gr-A549 cells (scale bars: 200 µm and 100 µm, respectively). (**B**): Relative expression of the stemness-associated genes SOX2, OCT4, and NANOG in CSLCs and Gr-CSLCs, with parental A549 cells as the reference group. (**C**): Flow cytometric characterization of CSLCs and Gr-CSLCs using CD326, CD133, CD44, CD90, ABCG2, and MDR1. Marker-positive populations and corresponding isotype controls are shown in purple and blue, respectively. Data represent three independent biological replicates (*n* = 3). Statistical analysis in B was performed using one-way ANOVA followed by Tukey’s HSD post hoc test; ** *p* < 0.01 indicates the significant comparison shown in the graph.

The A549 and gemcitabine-hydrochloride-resistant (Gr-A549) human non-small cell lung cancer (hNSCLC) cell lines, previously established in our study (Demir Cevizlidere et al., 2024) [23] and cryopreserved, were thawed and examined morphologically using phase-contrast microscopy (Figure 1A). The parental A549 cells exhibited a morphology characterized by star-shaped extensions and typical epithelial-like features (Figure 1A(a)), whereas the Gr-A549 cells displayed distinct, well-defined colony formations consisting of at least 50 viable cells, indicating a phenotypic alteration associated with the development of chemoresistance (Figure 1A(b)).

To assess the expression of pluripotency-associated transcription factors, we performed qRT-PCR analysis for SOX2, OCT4, and NANOG in thawed CSLC and Gr-CSLC spheroids. A549 parental cells served as the reference group. Our results revealed that OCT4 was only weakly expressed in CSLCs but exhibited a marked upregulation in Gr-CSLCs, indicating a possible association with the drug-resistant phenotype. In contrast, SOX2 and NANOG expression levels were comparable between CSLCs and Gr-CSLCs, suggesting that these stemness-associated markers remained relatively stable across the two populations (Figure 1B).

To verify that thawed CSLC and Gr-CSLC spheroids retained their stem-like immunophenotypic characteristics, surface marker expression was re-evaluated by flow cytometry. Both CSLC and Gr-CSLC populations expressed the evaluated lung cancer stem cell-associated markers, although differences in marker positivity were observed between the two populations. In CSLCs, marker positivity was CD326 (EpCAM) 63.42%, CD133 50.96%, CD44 99.99%, CD90 86.54%, ABCG2 (CD338) 52.86%, and MDR1 22.40%. In Gr-CSLCs, marker positivity was CD326 (EpCAM) 60.54%, CD133 71.65%, CD44 100.00%, CD90 88.99%, ABCG2 (CD338) 56.57%, and MDR1 54.11% (Figure 1C). Notably, Gr-CSLCs showed higher CD133 and MDR1 positivity than CSLCs, with the most pronounced difference observed for MDR1. These findings support the preservation of stem-like marker expression in the CSLC populations following the freeze–thaw process. Additionally, Gr-CSLCs showed markedly higher MDR1 positivity than CSLCs (54.11% vs. 22.40%, respectively), consistent with their drug-resistant phenotype.

**Figure 2:**
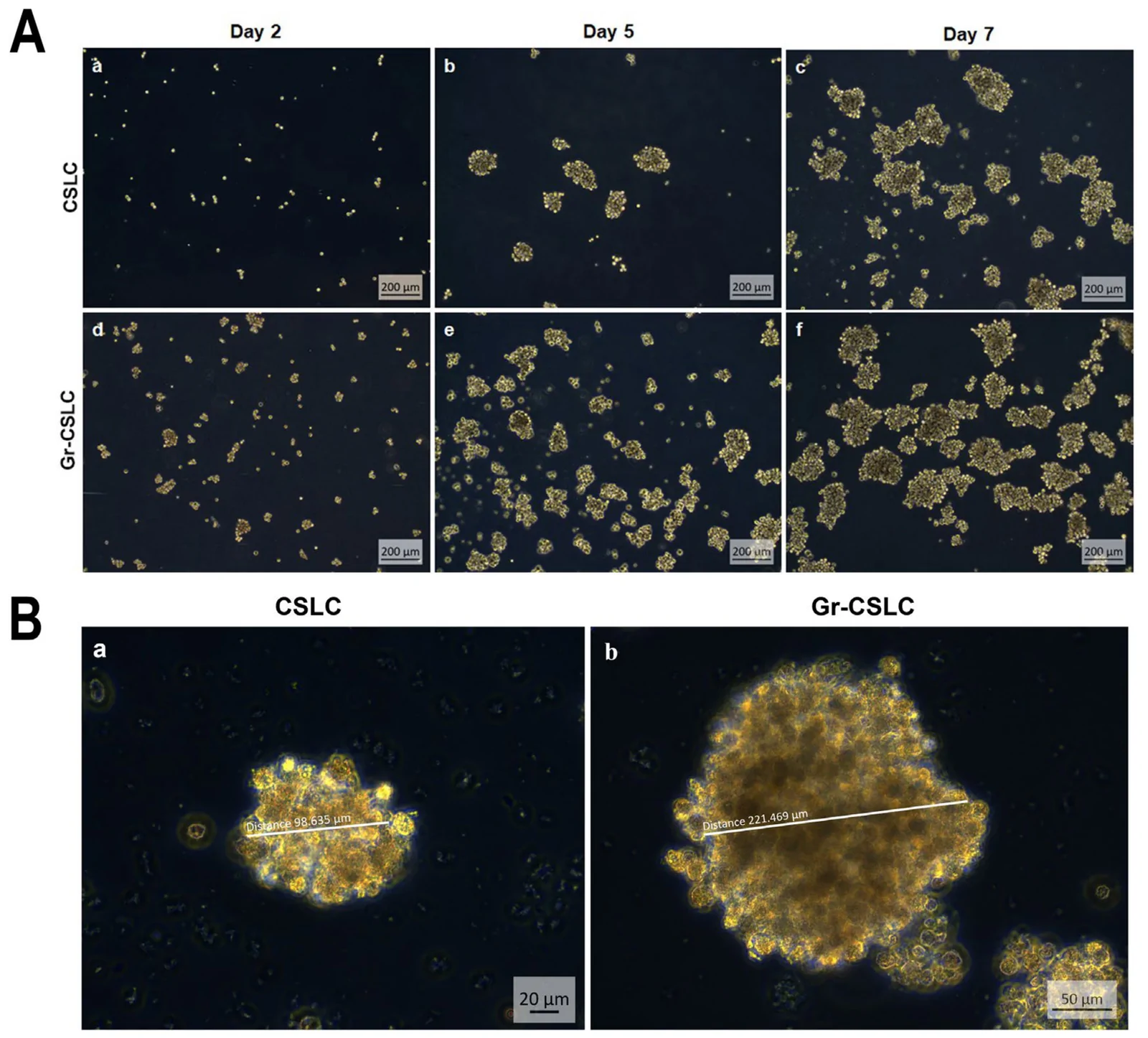
Spheroid-forming characteristics of CSLCs and Gr-CSLCs. (**A**) Phase-contrast microscopic images of spheroids generated on Poly-D-HEMA-coated plates: (**a**–**c**) CSLC spheroids at days 2, 5, and 7, respectively; (**d**–**f**) Gr-CSLC spheroids at days 2, 5, and 7, respectively (scale bars: 200 µm). (**B**) Representative phase-contrast microscopic images of spheroids at day 7: (**a**) CSLC spheroid and (**b**) Gr-CSLC spheroid, showing the larger spheroid size observed in the Gr-CSLC culture (scale bars: 20 µm and 50 µm, respectively).

Following thawing and culture of previously cryopreserved CSLC and Gr-CSLC spheroids, their tumorsphere-forming abilities were re-evaluated under serum-free, non-adherent conditions for a 10-day period. Spheroid formation became evident starting from day 5 in CSLCs and as early as day 3 in Gr-CSLCs, with mature spheroids appearing by days 7 and 5, respectively (Figure 2A). The spheroids were morphologically uniform, ranging from 100 to 250 μm in diameter. Gr-CSLCs demonstrated earlier and more robust spheroid formation than CSLCs, and larger spheroids were observed in the Gr-CSLC cultures at day 7 (Figure 2B), consistent with a more proliferative and self-renewing phenotype.

**Figure 3:**
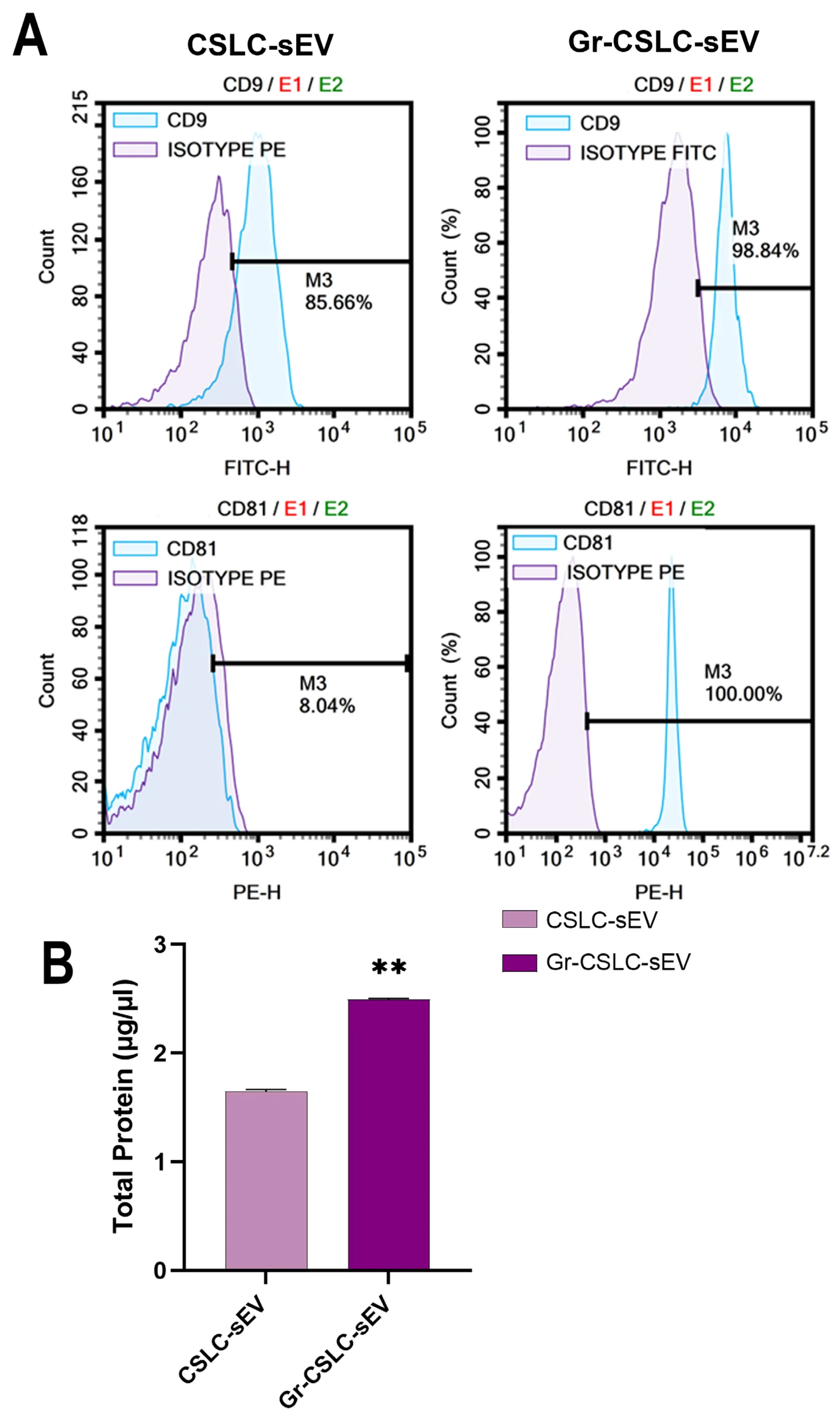
Characterization of sEV preparations isolated from CSLC and Gr-CSLC spheroids. (**A**): Immunophenotypic flow cytometric analysis of the EV-associated tetraspanins CD9 and CD81 in CSLC- and Gr-CSLC-derived sEV preparations. The reported quantitative values represent the mean percentage positivity obtained from three independent biological replicates (*n* = 3 per group). (**B**): Total protein content of CSLC- and Gr-CSLC-derived sEV preparations normalized to the initial cell number. Significance level is indicated as ** *p* < 0.01.

To characterize EV-associated surface markers in the isolated sEV preparations, flow cytometry was performed following magnetic bead-based capture using CD63-coated beads. Staining with fluorochrome-conjugated antibodies against the EV-associated tetraspanins CD9 (PE) and CD81 (FITC) demonstrated detectable tetraspanin expression in the CSLC- and Gr-CSLC-derived sEV preparations (Figure 3A). Across three biological replicates (*n* = 3), mean CD9 positivity was 85.66% in CSLC-derived sEV preparations and 98.84% in Gr-CSLC-derived sEV preparations, whereas mean CD81 positivity showed a marked difference between the two groups (8.04% and 100.00%, respectively) (Figure 3A).

The total protein content of sEV preparations isolated from CSLC and Gr-CSLC spheroids was measured using the Qubit™ Protein Assay Kit. For an equal input of 1 × 10^7^ cells, protein concentrations were 1.63 μg/μL for CSLC-derived sEV preparations and 2.48 μg/μL for Gr-CSLC-derived sEV preparations (Figure 3B). Thus, the Gr-CSLC-derived sEV preparation showed approximately 1.5-fold higher total protein content than the CSLC-derived sEV preparation.

**Figure 4:**
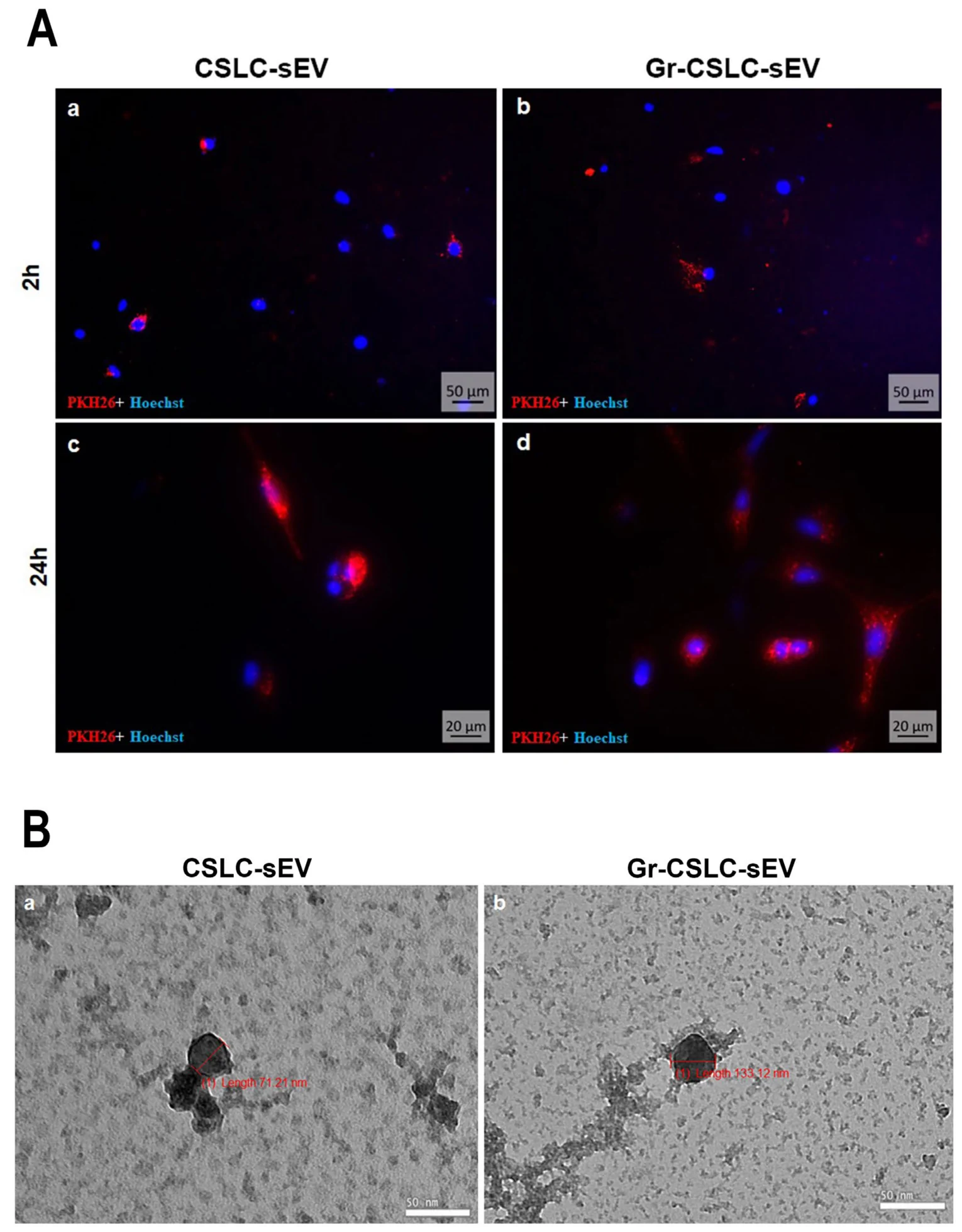
PKH26-based cellular uptake assessment and cryo-TEM characterization of CSLC- and Gr-CSLC-derived sEV preparations. (**A**) Fluorescence microscopy images of A549 cells following exposure to PKH26-labeled sEV preparations: (**a**) CSLC-derived sEVs at 2 h; (**b**) Gr-CSLC-derived sEVs at 2 h; (**c**) CSLC-derived sEVs at 24 h; and (**d**) Gr-CSLC-derived sEVs at 24 h. Red fluorescence represents PKH26-associated signals, and nuclei were counterstained with Hoechst 33342 (blue) (scale bars: 50 µm in (**a**,**b**) and 20 µm in (**c**,**d**)). (**B**) Cryogenic transmission electron microscopy (cryo-TEM) images showing vesicular structures in (**a**) CSLC-derived and (**b**) Gr-CSLC-derived sEV preparations (scale bar: 50 nm).

To assess the cellular uptake of CSLC- and Gr-CSLC-derived sEV preparations, A549 human lung carcinoma cells were incubated with PKH26-labeled sEVs. Fluorescence microscopy showed intracellular/perinuclear PKH26-associated red signals in A549 cells after 2 and 24 h of exposure, with stronger signals observed at 24 h. These findings are consistent with uptake-associated accumulation of labeled sEV material in recipient cells (Figure 4A).

Cryogenic transmission electron microscopy (cryo-TEM) demonstrated round-to-oval, membrane-bound vesicles in CSLC- and Gr-CSLC-derived sEV preparations. Representative vesicle diameters ranged from approximately 70 to 140 nm, and measurements were obtained from at least 10 vesicles per group. These ultrastructural observations were consistent with the presence of small extracellular vesicles (sEVs) (Figure 4B).

**Figure 5:**
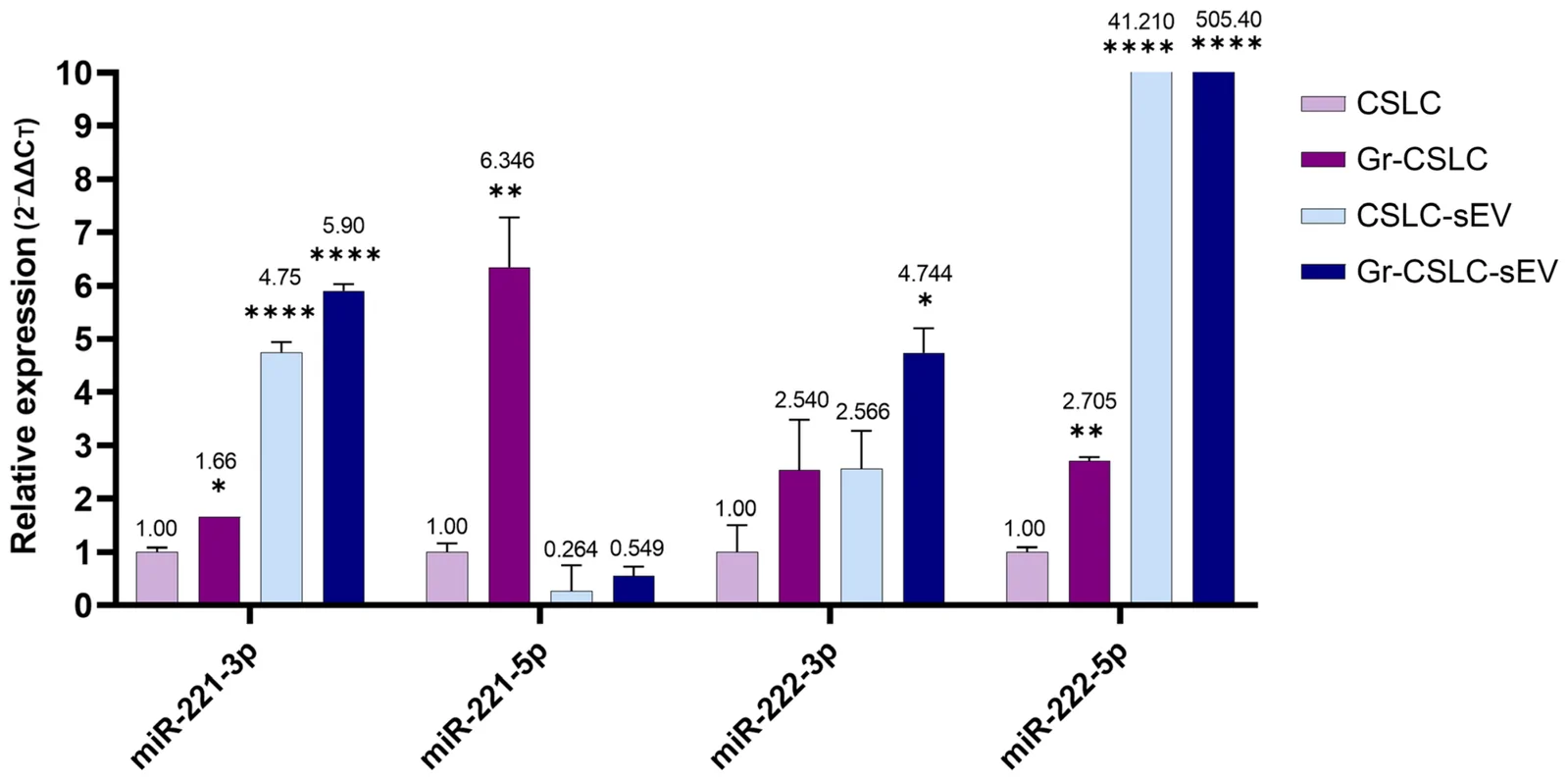
qRT-PCR analysis of miR-221/222 family members in CSLCs, Gr-CSLCs, and their corresponding sEVs. Relative expression levels of miR-221-3p, miR-221-5p, miR-222-3p, and miR-222-5p were normalized to U6 snRNA using the 2^−^ΔΔCt method, with CSLCs as the calibrator group. Data represent three independent biological replicates (*n* = 3). Statistical analysis was performed using one-way ANOVA followed by Tukey’s HSD post hoc test. Significance levels are indicated as * *p* < 0.05, ** *p* < 0.01, and **** *p* < 0.0001.

Quantitative real-time PCR analysis was performed to assess the expression levels of miR-221/222 cluster members (hsa-miR-221-3p, hsa-miR-221-5p, hsa-miR-222-3p, and hsa-miR-222-5p), along with drug resistance-associated genes (MDR1, ABCG2, and BCL2) in CSLCs, Gr-CSLCs, and their corresponding sEVs. Among the miRNAs analyzed, miR-221-3p, miR-222-3p, and miR-222-5p were significantly upregulated in Gr-CSLC-derived sEVs compared with CSLC-derived sEVs, whereas miR-221-5p showed no significant difference between these two groups (Figure 5).

**Figure 6:**
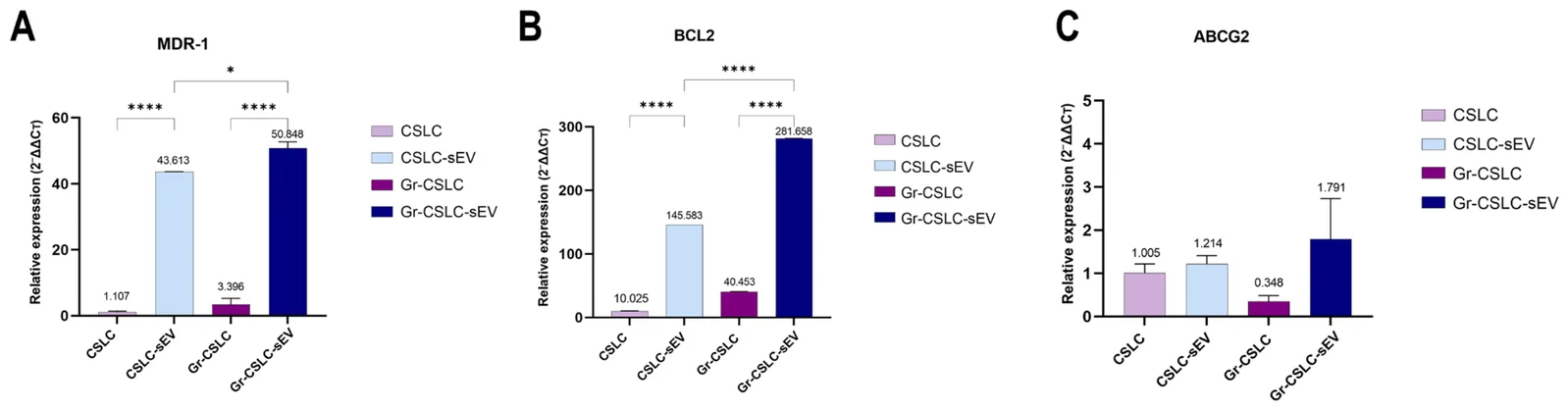
qRT-PCR analysis of multidrug resistance-associated genes in CSLCs, Gr-CSLCs, and their corresponding sEVs. Relative expression levels of MDR1/ABCB1 (**A**), BCL2 (**B**), and ABCG2 (**C**) were normalized to GAPDH using the 2^−^ΔΔCt method, with CSLCs as the calibrator group. Data represent three independent biological replicates (*n* = 3). Statistical analysis was performed using one-way ANOVA followed by Tukey’s HSD post hoc test. Significance levels are indicated as * *p* < 0.05, and **** *p* < 0.0001.

Regarding mRNA expression, MDR1 and BCL2 transcripts showed higher relative expression in Gr-CSLCs and Gr-CSLC-derived sEVs compared with their non-resistant counterparts (Figure 6A,B). However, ABCG2 expression levels remained comparable across CSLCs, Gr-CSLCs, and their respective sEVs, with no statistically significant differences detected (Figure 6C). These findings indicate higher relative levels of specific miR-221/222 members—particularly miR-221-3p, miR-222-3p, and miR-222-5p—in Gr-CSLC-derived sEVs than in CSLC-derived sEVs, consistent with molecular differences associated with the drug-resistant phenotype. The observed MDR1 and BCL2 expression profiles further distinguish sEV preparations derived from gemcitabine-resistant Gr-CSLCs from those derived from gemcitabine-sensitive CSLCs.

## 4. Discussion

In the initial phase of this study, previously isolated, characterized and cryopreserved CSLC and Gr-CSLC lines from our ESTEM laboratories [23] were re-evaluated to ensure phenotypic stability, viability and functional competence after freeze–thaw. Trypan blue exclusion assays confirmed high viability in both populations, consistent with reports that cryopreservation preserves stemness and viability in CSCs [24,25,26]. Flow cytometry demonstrated sustained expression of canonical CSC markers—CD326 (EpCAM), CD133, CD44, CD90, ABCG2 (CD338) and ALDH1—in both CSCs and Gr-CSCs, in line with standard CSC profiles described in multiple tumor models [27,28]. Importantly, MDR1—a key chemoresistance marker—was significantly upregulated in Gr-CSLCs, reflecting the preserved resistant phenotype initially induced in the parental Gr-A549 line and consistent with MDR1-driven resistance in stem-like cancer cells [7]. qRT-PCR showed comparable SOX2 and NANOG expression in CSLC and Gr-CSLC spheroids, whereas OCT4 expression was markedly higher in Gr-CSLCs. This finding is consistent with previous evidence linking elevated OCT4 expression to the maintenance of cancer stem-like properties and therapy-resistant phenotypes, including in lung cancer [29,30,31]. Interestingly, the selective increase in OCT4 in the absence of corresponding changes in SOX2 and NANOG suggests that the resistant Gr-CSLC phenotype was not associated with a uniform upregulation of all pluripotency-related transcription factors. Rather, OCT4 may represent a more selectively altered stemness-associated feature in this model; however, because the present study did not manipulate OCT4 expression or directly assess its downstream signaling, a causal contribution of OCT4 to gemcitabine resistance cannot be established. Functionally, both cell lines retained robust spheroid-forming capacity under serum-free conditions, with Gr-CSLCs generating larger spheroids more rapidly—an indication of a more aggressive growth phenotype [32,33]. The spheroid-based approach is particularly relevant to the present study because it allowed sEV-associated molecular profiles to be examined in CSLC-enriched three-dimensional cultures rather than conventional monolayer tumor-cell cultures. Accordingly, comparing sEVs derived from gemcitabine-sensitive and -resistant CSLC spheroids provided a controlled experimental framework for identifying resistance-associated differences in vesicular RNA profiles within a stem-like tumor-cell context. Together, these data confirm that cryopreserved CSLC and Gr-CSLC lines maintain their phenotypic, molecular, and functional stem-like characteristics and provide a suitable experimental model for investigating sEV-associated molecular features linked to gemcitabine resistance [8,9].

To ensure the reliability of downstream molecular analyses, exosomes derived from CSLC and Gr-CSLC spheroids were comprehensively characterized for protein content, surface markers, morphology and uptake by recipient cells. The higher total protein content observed in Gr-CSLC-derived sEV preparations compared with CSLC-derived sEV preparations indicates a difference in the recovered vesicular preparations; however, in the absence of particle concentration measurements, this finding cannot be interpreted as evidence of increased sEV secretion or production. Alterations in EV secretion and molecular cargo have been reported in other chemoresistant cancer models and have been associated with changes in paracrine signaling within the tumor microenvironment [13,34,35,36]. Bead-based flow cytometry demonstrated the presence of EV-associated tetraspanins in the isolated sEV preparations, providing additional evidence for their vesicular characterization [12,28,37,38]. Notably, mean CD81 positivity differed markedly between CSLC- and Gr-CSLC-derived sEV preparations (8.04% vs. 100.00%, respectively). However, given the absence of replicate-level flow cytometric measurements and fluorescence-intensity data, the biological significance of this difference cannot be established from the present dataset and should therefore be interpreted cautiously. Cryo-TEM showed spherical, membrane-bound vesicles of 70–140 nm in diameter, without aggregation or deformation, matching the expected size range and morphology of exosomes [11,39]. PKH26-labeling experiments showed intracellular perinuclear fluorescence in A549 cells following exposure to CSLC- and Gr-CSLC-derived sEV preparations. However, because dye-only, vehicle, and unlabeled-sEV controls were not included in the original uptake experiment, the observed PKH26-associated fluorescence cannot be attributed exclusively to internalized sEVs and should therefore be interpreted cautiously as uptake-associated fluorescence rather than definitive evidence of vesicle internalization. Previous studies have reported cellular uptake of CSC-derived extracellular vesicles and their potential to carry bioactive molecules, including miRNAs and drug-resistance-related proteins [14,40]; however, such functional transfer was not directly demonstrated in the present study. Overall, the protein content, tetraspanin expression, ultrastructural morphology, and uptake-associated fluorescence support the presence of small extracellular vesicles (sEVs); however, these approaches do not conclusively establish their endosomal origin.

One of the prominent findings of this study was the significantly higher relative expression of miR-221-3p, miR-222-3p, and miR-222-5p in sEVs derived from gemcitabine-resistant Gr-CSLCs compared with sEVs derived from gemcitabine-sensitive CSLCs. Among these, miR-222-5p showed the most pronounced difference, identifying it as a candidate for further investigation in the context of gemcitabine resistance. This pattern is consistent with reports implicating the miR-221/222 cluster—especially miR-222-5p—in tumor aggressiveness and therapy resistance across multiple malignancies, including hepatocellular carcinoma, breast cancer and NSCLC [10,19,41,42]. Upregulation of the cluster has been associated with enhanced proliferation, migration and resistance to apoptosis through modulation of key pathways such as PTEN/AKT and ERα signaling [20,21,22]. Our data extend this literature by showing that differences in miR-221/222 expression associated with the resistant Gr-CSLC phenotype are also reflected in their sEV-associated RNA profiles; however, the present findings do not establish functional transfer of chemoresistance to recipient cells.

We further observed that this sEV-associated miRNA profile was accompanied by higher relative expression of MDR1 (ABCB1) and BCL2 in Gr-CSLC-derived sEVs. MDR1 and BCL2 are central mediators of multidrug resistance and apoptosis evasion, respectively [7,43]. The concurrent detection of higher relative levels of miR-222-5p, MDR1, and BCL2 in Gr-CSLC-derived sEV preparations identifies an association with the resistant phenotype, but does not establish their co-packaging, synergistic interaction, or functional contribution to gemcitabine resistance. Co-packaging of resistance-related mRNAs and miRNAs in exosomes has been described in pancreatic and colorectal cancer and associated with horizontal transfer of chemoresistance [28,34,35]. In contrast, ABCG2 expression did not differ significantly among CSLCs, Gr-CSLCs, and their corresponding sEV preparations, indicating that the expression pattern observed for MDR1 was not shared by all multidrug resistance-associated genes examined in this study. This observation is consistent with reports that the relative contribution of individual ABC transporters may vary according to drug and tumor context [44].

Previous studies have shown that tumor-derived extracellular vesicles can mediate intercellular communication through the transfer of bioactive molecules, including miRNAs, providing a biological framework for their potential involvement in cancer-associated phenotypes and treatment resistance [14,15]. However, extracellular vesicle-mediated intercellular communication represents only one component of the multifactorial mechanisms underlying acquired chemoresistance. Drug resistance can also arise through multiple cell-intrinsic and microenvironment-associated mechanisms, including alterations in drug transport and metabolism, DNA-damage responses, apoptosis and survival signaling, epithelial–mesenchymal plasticity, and the persistence of stem-like tumor-cell populations [7,44]. Thus, the sEV-associated molecular differences observed in the present study should be considered within this broader network of resistance mechanisms rather than as evidence that sEV-mediated communication is the sole route through which gemcitabine resistance is acquired or maintained. Collectively, these findings demonstrate distinct molecular profiles in sEV preparations derived from gemcitabine-resistant Gr-CSLCs compared with those derived from gemcitabine-sensitive CSLCs, particularly with respect to miR-221/222 family members, MDR1, and BCL2. The detection of PKH26-associated intracellular/perinuclear fluorescence following exposure of A549 cells to these preparations is consistent with cellular uptake of labeled vesicular material; however, the present study did not directly determine whether sEV-associated RNAs were functionally transferred to recipient cells or whether such exposure altered gemcitabine sensitivity, apoptosis, or cell survival. Therefore, the observed sEV-associated molecular differences should be interpreted as correlates of the resistant phenotype rather than evidence of functional transmission of chemoresistance. More broadly, extracellular vesicle-mediated intercellular communication has been implicated in tumor progression and adaptation within the tumor microenvironment [12,13]. The higher relative level of miR-222-5p observed in Gr-CSLC-derived sEVs also identifies this miRNA as a candidate for further investigation as a resistance-associated biomarker; however, its predictive or diagnostic utility in NSCLC will require validation in patient-derived samples and clinical cohorts [16,18,45].

From a translational perspective, the higher relative levels of miR-221/222 family members, particularly miR-222-5p, together with MDR1 and BCL2 in Gr-CSC-derived sEV preparations identify these molecules as candidates for further investigation in the context of gemcitabine resistance. If validated in patient-derived samples and biofluids, such sEV-associated molecular signatures could potentially contribute to the development of minimally invasive approaches for monitoring treatment-associated molecular changes. From a therapeutic perspective, determining whether sEV-associated miR-221/222 family members and resistance-related transcripts such as MDR1 and BCL2 functionally contribute to the resistant phenotype could identify components of EV-mediated signaling as potential intervention targets. Future studies could therefore investigate whether modulation of these candidate cargo molecules, interference with their EV-mediated transfer, or disruption of their downstream resistance-associated pathways can restore or enhance gemcitabine sensitivity. Such strategies would require direct functional validation in recipient-cell assays and subsequent confirmation in additional NSCLC models and patient-derived systems before any therapeutic relevance can be established. However, the present study did not assess clinical specimens, biomarker performance, or functional transfer of gemcitabine resistance; therefore, these potential diagnostic and therapeutic applications remain to be established in future studies [16,18,40,46].

Despite the novelty and strengths of this work, several limitations should be acknowledged. First, although the use of isogenic CSLC and Gr-CSLC populations enabled a tightly controlled comparison of gemcitabine-sensitive and -resistant phenotypes, our analyses were restricted to a single A549-derived NSCLC model. Validation in additional NSCLC cell lines and patient-derived models will be necessary to determine the generalizability and clinical relevance of these findings. Second, although intracellular/perinuclear PKH26-associated fluorescence was observed in A549 cells following exposure to CSLC- and Gr-CSLC-derived sEV preparations, dye-only, vehicle, and unlabeled-sEV controls were not included in the original uptake experiment. Therefore, the observed PKH26-associated fluorescence cannot be attributed exclusively to internalized sEVs and should be interpreted cautiously. In addition, the downstream functional consequences of exposure to these preparations—such as changes in gemcitabine sensitivity, apoptosis, cell survival, or gene expression—were not directly evaluated. Future functional transfer studies, including appropriately controlled recipient-cell experiments and subsequent assessment of drug sensitivity and survival-related outcomes, will be required to determine whether Gr-CSLC-derived sEVs can functionally influence the chemoresistant phenotype of recipient cells. Third, the present study focused on a targeted panel of resistance- and stemness-associated molecules; broader, unbiased profiling of sEV-associated RNA and protein cargo by RNA sequencing and proteomic approaches could reveal additional molecular pathways and candidate therapeutic targets beyond the miR-221/222 cluster. In addition, U6 snRNA and GAPDH were used as reference genes for miRNA and mRNA normalization, respectively; however, their stability across cellular and extracellular vesicle samples was not independently validated. Accordingly, cross-compartment comparisons between cells and vesicles should be regarded as exploratory and interpreted cautiously. Finally, although cryo-TEM and tetraspanin analysis supported the presence of small membrane-bound extracellular vesicles, particle concentration and population-level size-distribution measurements, EV negative-marker analyses, and particle-to-protein ratios were not available in the present study. Therefore, the preparations are conservatively referred to as sEVs, and complementary particle-based approaches such as nanoparticle tracking analysis or tunable resistive pulse sensing, together with additional positive and negative EV markers, would be required for more comprehensive characterization in accordance with current EV guidelines [47].

In summary, our findings demonstrate that gemcitabine-resistant CSLCs derived from A549 not only retain their stem-like features following cryopreservation but also secrete exosomes enriched in miR-221/222 and resistance-associated transcripts such as MDR1 and BCL2. These vesicles, which are efficiently internalized by naïve A549 cells, may contribute to the intercellular dissemination of chemoresistance. Beyond their biomarker potential, the selective enrichment of functional cargo in Gr-CSLC-derived sEVs highlights the feasibility of targeting these vesicles or their miRNA content as modulators of resistance. Future work should validate these molecular signatures in patient-derived xenografts and clinical specimens and explore precision approaches, including exosomal cargo editing, inhibition of vesicle release or selective blockade of miRNA-mediated signaling within the tumor microenvironment. By addressing the non-cell-autonomous dimensions of resistance, such strategies may help overcome therapeutic failure and improve outcomes in hNSCLC.

## Figures and Tables

**Table 1:** qRT-PCR primers used in this study.

Gene	Sequences	Size (bp)	Tm (°C)
miR-221-3p	F: AGCUACAUUGUCUGCUGGGUUUC R: CGAGGAAGAAGACGGAAGAAT	- -	- -
miR-221-5p	F: ACCUGGCAUACAAUGUAGAUUU R: CGAGGAAGAAGACGGAAGAAT	- -	- -
miR-222-3p	F: AGCUACAUCUGGCUACUGGGU R: CGAGGAAGAAGACGGAAGAAT	- -	- -
miR-222-5p	F: CUCAGUAGCCAGUGUAGAUCCU R: CGAGGAAGAAGACGGAAGAAT	- -	- -
BCL2	F: TTGACAGAGCATGCTGTACTT R: ATCTTTATTTCATGAGGCACGTT	24 23	53.97 49.93
MDR1	F: TTGCTGCTTACATTCAGGTTTCA R: AGCCTATCTCCTGTCGCATTA	19 20	52.40 51.78
ABCG2	F: TTCCACGATATGGATTTACGG R: GTTTCCTGTTGCATTGAGTCC	21 21	50.45 52.40
SOX-2	F: TTGCTGCCTCTTTAAGACTAGGA R: TAAGCCTGGGGCTCAAACT	23 19	53.49 51.09
OCT-4	F: CTTGAATCCCGAATGGAAAGGG R: CCTTCCCAAATAGAACCCCCA	22 21	54.84 54.36
NANOG	F: TTTGTGGGCCTGAAGAAAACT R: AGGGCTGTCCTGAATAAGCAG	21 21	50.45 54.36
GAPDH	F: GAACGGGAAGCTGGTCATC R: TTGATGTTGGCGGGGATCT	22 20	56.70 53.83
U6	F: GCTTCGGCA GCAATACTAAAT R: CGCTTCACGAATTTGCGTGTCAT	25 23	54.40 55.27

## Data Availability

The raw data supporting the conclusions of this article will be made available by the authors on request.

## References

[1] Bray F., Ferlay J., Soerjomataram I., Siegel R.L., Torre L.A., Jemal A. (2018). Global cancer statistics 2018: GLOBOCAN estimates of incidence and mortality worldwide for 36 cancers in 185 countries. CA-Cancer J. Clin..

[2] Zhang H., Zhang L., Chen K., Chen G. (2022). Efficacy and safety of nivolumab for advanced/recurrent non-small-cell lung cancer: An up-to-date meta-analysis of large-scale phase III randomized controlled trials. Future Oncol..

[3] Pandit B., Royzen M. (2022). Recent development of prodrugs of gemcitabine. Genes.

[4] Wong A., Soo R.A., Yong W.P., Innocenti F. (2009). Clinical pharmacology and pharmacogenetics of gemcitabine. Drug Metab. Rev..

[5] Li Q., Wu J., Chen S., Zhao Y. (2018). Cancer stem cells: A new target for cancer therapy. Histol. Histopathol..

[6] Ju F., Atyah M.M., Horstmann N., Gul S., Vago R., Bruns C.J., Zhao Y., Dong Q.Z., Ren N. (2022). Characteristics of the cancer stem cell niche and therapeutic strategies. Stem Cell Res. Ther..

[7] Vaidya F.U., Sufiyan Chhipa A., Mishra V., Gupta V.K., Rawat S.G., Kumar A., Pathak C. (2022). Molecular and cellular paradigms of multidrug resistance in cancer. Cancer Rep..

[8] Ishiguro T., Ohata H., Sato A., Yamawaki K., Enomoto T., Okamoto K. (2017). Tumor-derived spheroids: Relevance to cancer stem cells and clinical applications. Cancer Sci..

[9] Bordanaba-Florit G., Madarieta I., Olalde B., Falcón-Pérez J.M., Royo F. (2021). 3D cell cultures as prospective models to study extracellular vesicles in cancer. Cancers.

[10] Liu S.L., Sun P., Li Y., Liu S.S., Lu Y. (2019). Exosomes as critical mediators of cell-to-cell communication in cancer pathogenesis and their potential clinical application. Transl. Cancer Res..

[11] Sundararajan V., Sarkar F.H., Ramasamy T.S. (2018). The multifaceted role of exosomes in cancer progression: Diagnostic and therapeutic implications. Cell. Oncol..

[12] Han Q.F., Li W.J., Hu K.S., Gao J., Zhai W.L., Yang J.H., Zhang S.J. (2022). Exosome biogenesis: Machinery, regulation, and therapeutic implications in cancer. Mol. Cancer.

[13] Maia J., Caja S., Strano Moraes M.C., Couto N., Costa-Silva B. (2018). Exosome-based cell-cell communication in the tumor microenvironment. Front. Cell Dev. Biol..

[14] Tan S., Xia L., Yi P., Han Y., Tang L., Pan Q., Tian Y., Rao S., Oyang L., Liang J. (2020). Exosomal miRNAs in tumor microenvironment. J. Exp. Clin. Cancer Res..

[15] Hu C., Meiners S., Lukas C., Stathopoulos G.T., Chen J. (2020). Role of exosomal microRNAs in lung cancer biology and clinical applications. Cell Prolif..

[16] Li X., Li X., Zhang B., He B. (2022). The role of cancer stem cell-derived exosomes in cancer progression. Stem Cells Int..

[17] Sorop A., Constantinescu D., Cojocaru F., Dinischiotu A., Cucu D., Dima S.O. (2021). Exosomal microRNAs as biomarkers and therapeutic targets for hepatocellular carcinoma. Int. J. Mol. Sci..

[18] Wang X., Tian L., Lu J., Ng I.O. (2022). Exosomes and cancer—Diagnostic and prognostic biomarkers and therapeutic vehicle. Oncogenesis.

[19] Song Q., An Q., Niu B., Lu X., Zhang N., Cao X. (2019). Role of miR-221/222 in tumor development and the underlying mechanism. J. Oncol..

[20] Xiang C., Zhang Y., Zhang Y., Liu C., Hou Y., Zhang Y. (2020). lncRNA LEF1-AS1 promotes proliferation and induces apoptosis of non-small-cell lung cancer cells by regulating miR-221/PTEN signaling. Cancer Manag. Res..

[21] Liu B., Che Q., Qiu H., Bao W., Chen X., Lu W., Li B., Wan X. (2014). Elevated miR-222-3p promotes proliferation and invasion of endometrial carcinoma via targeting ERα. PLoS ONE.

[22] Yang X., Yang Y., Gan R., Zhao L., Li W., Zhou H., Wang X., Lu J., Meng Q.H. (2014). Down-regulation of miR-221 and miR-222 restrains prostate cancer cell proliferation and migration that is partly mediated by activation of SIRT1. PLoS ONE.

[23] Demir Cevizlidere B., Uysal O., Avci H., Gunes Bagis S., Semerci Sevimli T., Dincer M., Qomi Ekenel E., Kara S.G., Soykan M.N., Eker Sariboyaci A. (2024). Establishment, culture and characterization of gemcitabine hydrochloride-resistant human non-small cell lung carcinoma cell line derived cancer stem cells. Cell Biochem. Funct..

[24] Karimi-Busheri F., Zadorozhny V., Shawler D.L., Fakhrai H. (2010). The stability of breast cancer progenitor cells during cryopreservation: Maintenance of proliferation, self-renewal, and senescence characteristics. Cryobiology.

[25] Eini L., Naseri M., Karimi-Busheri F., Bozorgmehr M., Ghods R., Madjd Z. (2020). Primary colonospheres maintain stem cell-like key features after cryopreservation. J. Cell. Physiol..

[26] Aramini B., Masciale V., Grisendi G., Bertolini F., Maur M., Guaitoli G., Chrystel I., Morandi U., Stella F., Dominici M. (2022). Dissecting tumor growth: The role of cancer stem cells in drug resistance and recurrence. Cancers.

[27] Greve B., Kelsch R., Spaniol K., Eich H.T., Götte M. (2012). Flow cytometry in cancer stem cell analysis and separation. Cytom. A.

[28] Ramos E.K., Tsai C.F., Jia Y., Cao Y., Manu M., Taftaf R., Hoffmann A.D., El-Shennawy L., Gritsenko M.A., Adorno-Cruz V. (2022). Machine learning-assisted elucidation of CD81-CD44 interactions in promoting cancer stemness and extracellular vesicle integrity. eLife.

[29] Chen Y.C., Hsu H.S., Chen Y.W., Tsai T.H., How C.K., Wang C.Y., Hung S.C., Chang Y.L., Tsai M.L., Lee Y.Y. (2008). Oct-4 expression maintained cancer stem-like properties in lung cancer-derived CD133-positive cells. PLoS ONE.

[30] Kim J.Y., Xue H., Lu L., Li Y., Li J. (2021). Role of OCT4 in cancer stem cells: Epigenetic regulation and therapeutic implications. Front. Oncol..

[31] Mohiuddin I.S., Wei S.J., Kang M.H. (2020). Role of OCT4 in cancer stem-like cells and chemotherapy resistance. Biochim. Biophys. Acta Mol. Basis Dis..

[32] Barbosa M.A.G., Xavier C.P.R., Pereira R.F., Petrikaitė V., Vasconcelos M.H. (2022). 3D cell culture models as recapitualators of the tumor microenvironment for the screening of anti-cancer drugs. Cancers.

[33] Edmondson R., Broglie J.J., Adcock A.F., Yang L. (2014). Three-dimensional cell culture systems and their applications in drug discovery and cell-based biosensors. Assay. Drug Dev. Technol..

[34] Lou G., Song X., Yang F., Wu S., Wang J., Chen Z., Liu Y. (2015). Exosomes derived from miR-122-modified adipose tissue-derived MSCs increase chemosensitivity of hepatocellular carcinoma. J. Hematol. Oncol..

[35] Fan P., Liu L., Yin Y., Zhao Z., Zhang Y., Amponsah P.S., Xiao X., Bauer N., Abukiwan A., Nwaeburu C.C. (2016). MicroRNA-101-3p reverses gemcitabine resistance by inhibition of ribonucleotide reductase M1 in pancreatic cancer. Cancer Lett..

[36] Sun Z., Wang L., Dong L., Wang X. (2018). Emerging role of exosome signalling in maintaining cancer stem cell dynamic equilibrium. J. Cell. Mol. Med..

[37] Théry C., Witwer K.W., Aikawa E., Alcaraz M.J., Anderson J.D., Andriantsitohaina R., Antoniou A., Arab T., Archer F., Atkin-Smith G.K. (2018). Minimal information for studies of extracellular vesicles 2018 (MISEV2018): A position statement of the International Society for Extracellular Vesicles and update of the MISEV2014 guidelines. J. Extracell. Vesicles.

[38] Wu Q., Qian W., Sun X., Jiang S. (2022). Small-molecule inhibitors, immune checkpoint inhibitors, and more: FDA-approved novel therapeutic drugs for solid tumors from 1991 to 2021. J. Hematol. Oncol..

[39] Tu J., Luo X., Liu H., Zhang J., He M. (2021). Cancer spheroids derived exosomes reveal more molecular features relevant to progressed cancer. Biochem. Biophys. Rep..

[40] Bhome R., Del Vecchio F., Lee G.H., Bullock M.D., Primrose J.N., Sayan A.E., Mirnezami A.H. (2018). Exosomal microRNAs (exomiRs): Small molecules with a big role in cancer. Cancer Lett..

[41] Wu Z.H., Tao Z.H., Zhang J., Li T., Ni C., Xie J., Zhang J.F., Hu X.C. (2016). MiRNA-21 induces epithelial to mesenchymal transition and gemcitabine resistance via the PTEN/AKT pathway in breast cancer. Tumour Biol..

[42] Amini S., Abak A., Sakhinia E., Abhari A. (2019). MicroRNA-221 and MicroRNA-222 in common human cancers: Expression, function, and triggering of tumor progression as a key modulator. Lab. Med..

[43] Makena M.R., Ranjan A., Thirumala V., Reddy A.P. (2020). Cancer stem cells: Road to therapeutic resistance and strategies to overcome resistance. Biochim. Biophys. Acta Mol. Basis Dis..

[44] Gebregiworgis T., Bhinderwala F., Purohit V., Chaika N.V., Singh P.K., Powers R. (2018). Insights into gemcitabine resistance and the potential for therapeutic monitoring. Metabolomics.

[45] Chen J., Hu C., Pan P. (2017). Extracellular vesicle microRNA transfer in lung diseases. Front. Physiol..

[46] Askenase P.W. (2022). Exosome carrier effects; Resistance to digestion in phagolysosomes may assist transfers to targeted cells; II transfers of miRNAs are better analyzed via systems approach as they do not fit conventional reductionist stoichiometric concepts. Int. J. Mol. Sci..

[47] Welsh J.A., Goberdhan D.C.I., O’Driscoll L., Buzas E.I., Blenkiron C., Bussolati B., Cai H., Di Vizio D., Driedonks T.A.P., Erdbrügger U. (2024). Minimal information for studies of extracellular vesicles (MISEV2023): From basic to advanced approaches. J. Extracell. Vesicles.

